# WSB1: from homeostasis to hypoxia

**DOI:** 10.1186/s12929-016-0270-3

**Published:** 2016-08-19

**Authors:** Moinul Haque, Joseph Keith Kendal, Ryan Matthew MacIsaac, Douglas James Demetrick

**Affiliations:** 1 Department of Pathology and Laboratory Medicine, University of Calgary, Calgary, AB T2N 4N1 Canada; 2 Department of Oncology, University of Calgary, Calgary, AB T2N 4N1 Canada; 3 Department of Medical Biochemistry, University of Calgary, Calgary, AB T2N 4N1 Canada; 4 Calgary Laboratory Services, Room 302, HMRB, 3330 Hospital Dr. N.W., Calgary, AB T2N 4N1 Canada

**Keywords:** WSB1, E3 ubiquitin ligase, Hypoxia, Cancer, HIPK2, VHL

## Abstract

The *wsb1* gene has been identified to be important in developmental biology and cancer. A complex transcriptional regulation of *wsb1* yields at least three functional transcripts. The major expressed isoform, WSB1 protein, is a substrate recognition protein within an E3 ubiquitin ligase, with the capability to bind diverse targets and mediate ubiquitinylation and proteolytic degradation. Recent data suggests a new role for WSB1 as a component of a neuroprotective pathway which results in modification and aggregation of neurotoxic proteins such as LRRK2 in Parkinson’s Disease, via an unusual mode of protein ubiquitinylation.

WSB1 is also involved in thyroid hormone homeostasis, immune regulation and cellular metabolism, particularly glucose metabolism and hypoxia. In hypoxia, *wsb1* is a HIF-1 target, and is a regulator of the degradation of diverse proteins associated with the cellular response to hypoxia, including HIPK2, RhoGDI2 and VHL. Major roles are to both protect HIF-1 function through degradation of VHL, and decrease apoptosis through degradation of HIPK2. These activities suggest a role for *wsb1* in cancer cell proliferation and metastasis. As well, recent work has identified a role for WSB1 in glucose metabolism, and perhaps in mediating the Warburg effect in cancer cells by maintaining the function of HIF1. Furthermore, studies of cancer specimens have identified dysregulation of *wsb1* associated with several types of cancer, suggesting a biologically relevant role in cancer development and/or progression.

Recent development of an inducible expression system for *wsb1* could aid in the further understanding of the varied functions of this protein in the cell, and roles as a potential oncogene and neuroprotective protein.

## Background

### Discovery and characterization of WSB1

The E3 ubiquitin ligase, WSB1, has been the subject of many investigations in diverse fields of biology that are finally starting to assemble the spectrum of functional activities attributed to this interesting protein. Aside from its initial characterization in developmental biology, recent studies show WSB1 has an intriguing role in the regulation of thyroid homeostasis, immune responsiveness, glycolysis and hypoxia, as well as potentially influencing the development or growth of cancer. Cell growth, survival and invasion have all been demonstrated to be influenced by WSB1. Recently, WSB1 has been implicated in a potentially important new neuroprotective pathway through its role as a mediator of protein ubiquitinylation.

### Identification and functional definition of *wsb1* as a developmental regulator

*wsb1* was originally identified in a virtual homology search by virtue of its relatedness to a large family of suppressor of cytokine signaling (SOCS)-box proteins, along with another related gene *wsb2* [15]. The proteins encoded a novel combination of known domains the WD40 repeats structurally located N-terminal to the SOCS box. The chick (*wsb1*) homologue was discovered as an early marker for limb bud development; likely to be involved in hedgehog pathway signalling [40]. The newly identified gene was initially named *SWiP-1* (SOCS box and WD repeats in Protein 1). Chick and human WSB1 (SWiP-1) have 88 % protein sequence similarity, while mouse and human WSB1 have 96.29 % sequence similarity [20, 40]. As such, the protein has been fairly conserved between distinct animal species. Whole mount in situ hybridization revealed that the *wsb1* expression pattern in the developing chick closely resembled to that of the hedgehog family of genes, specifically sonic hedgehog (*Shh*). High expression was found in the paraxial mesoderm (somites), limb buds, and other embryonic structures patterned by *Shh*. It was deduced that positive Shh signalling of *wsb1* was from the notochord, as blocking *Shh* expression in explant cultures depleted *wsb1* expression. A negative signal, possibly BMP4, was found from the intermediate and/or lateral mesoderm, preventing local *wsb1* expression.

The role of *wsb1* has been also studied in zebrafish development. The zebrafish WSB1 ortholog has 75 % protein sequence similarity with human WSB1 [25]. It was noted that *wsb1* transcript levels decreased during the mid-blastula transition (MBT) of the zebrafish embryo--an important turnover point of cell cycle regulation and gene expression. Injecting *wsb1* mRNA during this critical time resulted in morphological abnormalities and developmental arrest in zebrafish embryos. In summary, WSB1 activity served a significant role in the cell cycle regulation during zebrafish embryogenesis. Expression of the *wsb1* gene was found to be highly expressed in the intestine, heart, and spleen tissue in the adult zebrafish.

### WSB1 gene localization and expression

The International Radiation Hybrid Mapping Consortium mapped *wsb1* to chromosome 17 (G24371) of the human genome, on the *q* arm in proximity to the centromere (17q.11.1). The gene is 19.5 kilobases long. As reported in Ensembl, the gene encodes for 9 exons, and alternative splicing results in 17 putative transcripts (http://uswest.ensembl.org/Homo_sapiens/Gene/Summary?db=core;g=ENSG00000109046;r=17:27294076-27315926). However, only three major alternative *wsb1* transcripts have been reported in the literature [1, 3, 36]. It was shown, through Northern blotting of different human tissues, that the three *wsb1* mRNA were highly expressed in the brain, heart, kidney, and placenta [3]. Current review of the literature indicates that only isoform 1 of WSB1 has been detected at the protein level.

Many single nucleotide polymorphisms (SNP) have been identified both within coding and flanking regions of the gene in the human population (http://www.ncbi.nlm.nih.gov/projects/SNP/snp_ref.cgi?showRare=on&chooseRs=all&go=Go&locusId=26118). A very common SNP (heterozygosity 0.456, position 363) is present within exon 2. This SNP is a non-synonymous cytosine “C” to thymine “T” transition, creating a leucine to serine substitution (L16S) in the expressed protein. Structural biochemistry studies show that the presence of variants of this SNP within the *wsb1* RNA results in a difference to its secondary structure [43].

### Structure of WSB1

WSB1 was initially defined by eight WD repeats N-terminal to the C-terminal SOCS box [15, 40].

#### WD domain

This structural motif, containing approximately 40 amino acids comprised predominantly of tryptophan and aspartate dipeptide repeats, was first identified in bovine transducin β [14]. The WD motif defines one of the common domain structures in eukaryote proteins [38]. Structural analysis of the WD domain of β-transducin showed a 7-fold propeller-shaped structure with a core of conserved residues and the more variable linker regions displayed on the exterior face of each propeller blade [42]. The non-conserved stretches of amino acids located between β-strands form the surface of the propeller provide a framework for interactions with other proteins. The WD repeat number within a protein varies, and sometimes can be difficult to define. In the literature, researchers have reported different numbers of WD repeats for WSB1 [41]. Some groups have suggested six WD-repeat domains in WSB1 [44], some seven [10] and some eight [15, 40].

#### SOCS-Box

The SOCS box domain of WSB1 contains approximately 50 amino acids (1). It may possess a secondary structure composed of three α-helices [3, 42] as the region shows homology to that defining the members of the suppressors of cytokine signaling (SOCS) family of proteins [19]. More than 80 proteins containing the SOCS-box domain have been identified [24]. In almost all, including WSB1, the SOCS box can be located at the C terminus [15]. The SOCS box interacts with members of the E3 ubiquitin protein ligase complex such as Elongin B, C and the von-Hippel Lindau (VHL) tumor suppressor protein [18, 21, 49]. The SOCS-box consists of two main interaction regions—the BC box and the Cul box (reviewed in [24]). WSB1 contains the BC box [17]. Elongin C binds strongly to the BC site of the SOCS-box, whereas Elongin B stabilizes these interactions, however does not contact the SOCS-box [5]. Thus SOCS box proteins are important components of the ubiquitin E3 ligase complex.

Unfortunately, to date, the structure of WSB1 has not been determined at high resolution. Structural information of this protein would certainly add more clues with regards to its diversity of functions.

## Regulatory functions of WSB1

### Known WSB1 regulatory targets in humans

The first potential function for WSB1 was suggested from the ability of the BC-box containing WSB1 protein to interact with the CUL5 and RBX1 (cullin and ring-finger family members, respectively) proteins to form a potential ubiquitin ligase-E3 complex [17].

**Fig. 1:**
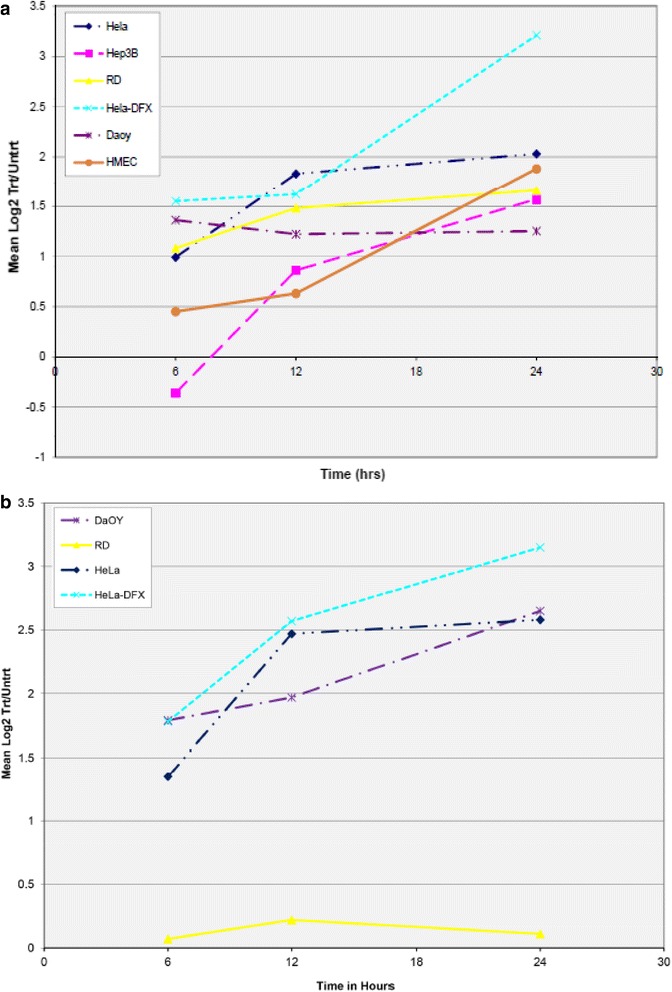
Regulation of WSB1 mRNA Expression by Hypoxia. Various cell lines including HeLa, Hep3B, RD, Daoy, and HMEC were treated with severe hypoxia for various time points (6, 12 and 24 h) as per methods published previously [34] and total mRNA extracted and evaluated by expression microarray. Panel **a** shows the normalized Log_2_(Treated/Untreated) ratios of EST AL110269 (*wsb1*) plotted for each time point. Cell lines are indicated by differing colors/lines. Panel **b** shows the normalized Log_2_(Treated/Untreated) ratios of EST AB018285 (JHDM2A/JMJD1A), a known HIF-1 target [31, 46] plotted for each time point. mRNA levels at Time_0_ are normalized to the Log_2_ value “0”. Cell lines are indicated by differing colors/lines. For both panels, Hela cells treated with 260uM desferroximine (Hela-DFX) was used as a positive control

**Fig. 2:**
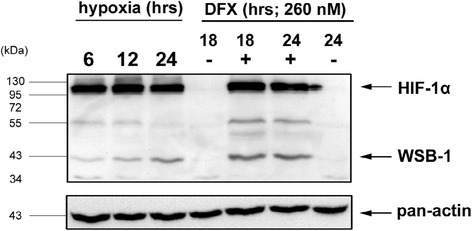
Regulation of WSB1 Protein Expression by Hypoxia. This figure shows a Western Blot of Hela cells treated with severe hypoxia or 260uM desferroximine for various timepoints (hours as indicated) and stained with either anti-pan-actin, anti-HIF1α or anti-WSB1 as per previously published methods [4]. The untreated (24 h−DFO) specimen is also the same as the Time_0_ hypoxia specimen as the experiments were performed simultaneously
    An initial role for WSB1 in hypoxia was first defined through its effect on the regulation of homeodomain-interacting protein kinase 2 (HIPK2) activity [9]. HIPK2 is a member of the nuclear protein kinase family and can induce cell death via the p53 and CtBP-mediated apoptotic pathways when activated [9, 26] under conditions such as DNA damage. HIPK2 phosphorylates p53 on S46, which upregulates its pro-apoptotic and cell cycle arrest activities. As such, HIPK2 concentrations are kept low in cells under normal physiological conditions--a process mediated by several proteins. WSB1 with its WD-repeats recognized and bound to HIPK2, which then led to HIPK2 ubiquitination and degradation. The group also demonstrated that WSB1 knockdown by RNA interference increased stability and durability of HIPK2 [9]. Therefore, in unstressed cells, WSB1 is a negative regulator of HIPK2 and is involved in maintaining homeostasis of HIPK2 activity.
    Investigation of the potential role of HIPK2 in hypoxia noted the surprising loss of HIPK2 protein. This was not mediated through decreased transcription, but through increased protein degradation. Initial experiments identified the E3-ligase, Siah1, as a mediator of this increased degradation, and further experiments also identified WSB1 as a mediator of hypoxia-associated HIPK2 degradation [26]. Interestingly, decreased levels of HIPK2 in hypoxia were associated with a decreased level of p53 S46 phosphorylation, and a blunted apoptotic response to the chemotherapeutic drug, adriamycin. These experiments suggest a mechanism by which cancer cells in a hypoxic environment can escape chemotherapy-associated (and maybe radiation-associated) apoptosis through the downregulation of HIPK2 [26]. Recently, VHL (von Hippel Lindau protein), the ubiquitin ligase responsive for targeted degradation of HIF-1α, has also been shown to be a target of WSB1, via functions similar to those mediating degradation of HIPK2 [22]. Thus, activity of WSB1 results in targeted ubiquitination and downregulation of VHL, leading to an increase in HIF-1 activity. The negative regulatory effect of WSB1 on VHL leads to an increase in transcription of HIF-1 targets and stimulates the cellular response to hypoxia, as well as other phenotypes of HIF-1 upregulation [22].
    Interestingly, *wsb1* disruption during hypoxia through RNAi silencing, or the loss of function of its E3 ubiquitin ligase, were both found to significantly suppress cell migration [6], which also reproduced the findings of other investigators with epithelial cells [22]. Upon further investigation, a strong negative correlation between WSB1 and a Rho-inhibiting protein (RhoGDI2) levels were found [6]. Functional studies confirmed that WSB1, using its SOCS box, directly ubiquitinylates RhoGDI2 and targets its degradation.
  - Glucose: HIF-1 has long been known to regulate genes involved in glucose metabolism (reviewed in [50]), as well as WSB1, and is a likely mediator of the well known Warburg effect of altered glucose metabolism in cancer cells. Recent work has identified that *wsb1*, which is a transcriptional target of HIF-1, is also target for the microRNA (miR) miR-592, which downregulates its expression [16]. Interestingly, miR-592 was identified to be commonly downregulated in hepatocellular carcinomas (HCCs), and that tumors with low expression of miR-592 tended to have a poor prognosis. Furthermore, upregulation of miR-592 decreased glucose metabolism in HCC cells and the growth of HCC tumor xenografts, and, conversely, downregulation of miR-592 was associated with increased HCC growth. Specimens of HCC from cancer patients showed a general inverse correlation between miR-592 levels and the levels of WSB1 and HIF1α. As WSB1 is a positive regulator of HIF-1 and miR-592 is a negative regulator of WSB-1, investigators have postulated that miR-592 is a negative mediator of the Warburg effect in HCC cells, and that the HIF-1/WSB1/miR-592 regulatory axis may be a new point of treatment for HCC cases [16].

Several physiologic targets of WSB1 activity have now been defined, representing different pathways of cellular homeostasis:

i. Thyroid homeostasis: WSB1 participated in an E3 complex to ubiquitinylate the metabolic regulatory protein, thyroid hormone-activating type 2 iodothyronine deiodinase(D2) [47]. D2 upregulates triiodothyronine (T3) concentration in developing tissues and in the adult brain, thereby controlling several physiological processes [2]. The WD-propeller domain of WSB1 recognized an 18 amino acid loop of D2 [48], while the SOCS domain mediated interaction with the E3 complex [10]. In the developing chick, sonic hedgehog-regulated WSB1 resulted in ubiquitination of D2 and chondrocyte growth, demonstrating a pathway by which sonic hedgehog mediated chick skeletal development [10]. D2 degradation stabilized parathyroid hormone-related peptide (PTHrP) production, an important factor for promoting chondrocyte proliferation [10, 33]. In the rat brain, WSB1 and the D2-deubiquitinase USP-33 expression were restricted to specific regions and worked in opposition to regulate the degradation of D2. Neither gene expression correlated the brain response to thyroid hormone, suggesting that the hormone does not control their regulation [13]. Moreover, correlation between (*wsb1*) and *USP-33* expression existed in other tissues not expressing D2. This could mean other ubiquitinylation substrates in those respected tissues may be similarly regulated by these antagonistic proteins. Recombinant cell line model systems have demonstrated that WSB1 interacts with ubiquitin conjugase UBC-7 in mediating D2 ubiquitination [33].
ii. Immune regulation: WSB1 plays a role in the maturation of the interleukin-21 receptor (IL-21R) [28]. The ligand of IL-21R is interleukin 21 (IL-21), a characterized cytokine with pleiotropic effects. IL-21 is known to regulate lymphocyte and myeloid cell activities. Through interaction with the cytoplasmic regions of IL-21R, WSB1 aided in its maturation—allowing for effective glycosylation [28]. By doing so, WSB1 moderated and decreased IL-21R degradation.
iii. Metabolic Regulation
  - Hypoxia: *wsb1* mRNA expression is upregulated in hypoxia at both mRNA and protein levels in diverse cell lines (Fig. 1a, b). Upregulation of *wsb1* is through the activity of HIF-1 [39]. Upregulation of *wsb1* can be achieved either through hypoxia chamber treatment or through a hypoxia mimic; such as the addition of desferroximine mesylate (DFX) (Fig. 2a, b), with a corresponding increase in HIF-1 activity [39].

### Protein aggregation

Several degenerative neurological disorders are characterized by the formation of protein aggregates, forming insoluble complexes or even microscopially visible macro-aggregates (“bodies”). More famous examples include the neurofibrillary tangles of Alzheimer’s dementia, as well as Lewy bodies, found in the dopaminergic neurons of the *substantia nigra* of Parkinson’s Disease (PD). Recently, one of the proteins commonly found in Lewy bodies, the kinase LRRK2, was found to be a target of WSB1 [30]. LRRK2 is known to be a mediator of a genetic PD syndrome, which is associated with gain of LRRK2 activity as a consequence of an activating mutation. WSB1 E3 ubiguitin ligase activity was shown to result in ubiquitinylation of LRRK2. Interestingly, though, instead of facilitating the conjugation of ubiquitin through the K48 residue, which is associated with proteosomal degradation, WSB1 facilitated the conjugation of ubiquitin residues K27 and K29 to LRKK2. This covalent association of ubiquitin with LRKK2 led to protein aggregation and loss of soluble LRKK2, rather than proteosomal degradation. Immunohistochemical study of Lewy bodies identified both WSB1 and LRRK2 within Lewy bodies associated with LRRK2-associated Parkinson’s Disease, but not PD associated with a different etiology. Furthermore, overexpression of *wsb1* led to significantly decreased neuronal toxicity due to upregulated LRRK2, while decreased *wsb1* expression led to increased neuronal toxicity. The authors hypothesize that these aggregates of insoluble, neurotoxic proteins are a cellular protective mechanism mediated by E3 proteins such as WSB1 [30].

### WSB1 compared to WSB2

WSB1 is related to WSB2 with an amino acid sequence similarity of 65 % [15]. Surprisingly, WSB1 and WSB2 have both similar and conflicting functional properties. In contrast to WSB1, WSB2 was found to be a negative regulator of IL-21R expression and IL-21-induced signal transduction [27]. Overexpression of *wsb2* reduced IL-21R expression whereas siRNA targeting of *wsb2* enhances IL-21R expression [27]. Expression of *wsb2* was found in mouse embryonic and adult gonads [35]. *wsb2* expression persisted only in male embryos, suggesting that it had a male specific role in gonad development [35]. Ihh signalling inhibitors led to a downregulation of *wsb2* mRNA. Based on these results, Sarraj et al. (2007) concluded that *wsb2* could be a potential consequence of hedgehog signalling similar to *wsb1* [35]. (*wsb2*) has also been found to regulate the expression of the granulocyte colony-stimulating factor (G-CSF) receptor. WSB2 binding reduced the proliferative signalling of G-CSF required for neutrophil development [12].

## WSB1 and cancer

As E3 ubiquitin ligases mediate the regulated degradation of various regulatory proteins crucial for cell survival, many are candidate oncoproteins or tumor suppressor proteins. E3 ubiquitin ligases such as MDM2 are known oncoproteins [7], whereas others such as VHL are known tumor suppressor proteins [32]. Classification of which role for the E3 ubiquitin ligase depends on the role of their major target protein. In addition, members of the same family can have either function, such as the F-box family of proteins [45]. Reviews of SOCS-box proteins are complex, due to the diversity of proteins that harbor this motif responsible for interaction with Elongin B and C, but whose target association may be determined by different domains such as WD40 or ankyrin. For example, SH2-containing SOCS proteins (eg. SOCS1-7) likely function as tumor suppressor proteins [11]. In this context, downregulation or decreased function of WSB1 would be expected in cancer cells, however, participation of WSB1 in ubiquitin-mediated degradation of a tumor suppressor gene such as VHL may also demonstrate a role for WSB1 as an oncoprotein.

**Fig. 3:**
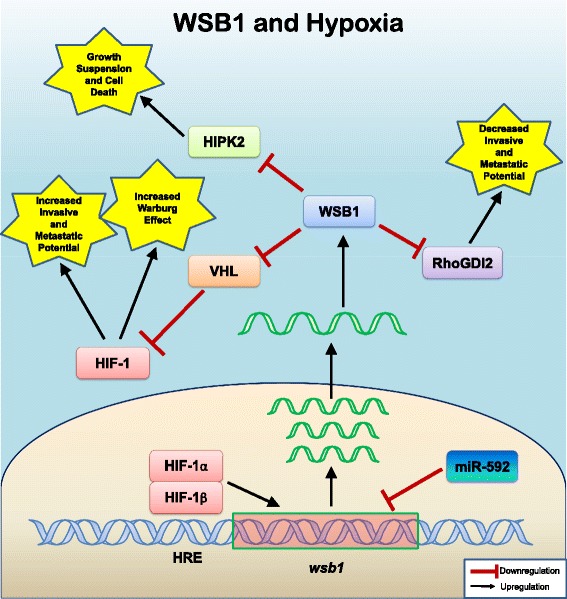
WSB1 has Diverse Roles in Hypoxia That May Also Drive Cancer Cell Growth. This figure summarizes the varied roles that WSB1 performs in the cellular response to hypoxia in cancer cells. In the normal hypoxic response, low oxygen results in inhibition of the VHL protein, which normally facilitates the degradation of the alpha subunit of the main hypoxia transcription factor HIF-1. HIF-1 upregulates a wide variety of target genes through interaction with its consensus Hypoxia Response Element, including *wsb1*, and a variety of other genes which can lead to enhanced cellular survival and metastasis in cancer cells. Upregulation of *wsb1* results in enhanced degradation of the VHL protein through WSB1, and increased activity of HIF-1, as well as increased degradation of other WSB1 targets HIPK2 and RhoDGI2, the effects of which can also stimulate cancer cell growth and metastasis. Aside from upregulation of the many HIF-1 target genes associated with the cellular response to hypoxia, HIF-1 also upregulates many of the genes whose products mediate the Warburg effect of glucose metabolism in cancer cells. The microRNA, miR-592, downregulates WSB-1 and thus can modulate the Warburg effect and the response to hypoxia. In summary, recent characterization of diverse roles for WSB1 in the cellular response to hypoxia suggests that WSB1 may function as an oncogene, leading to enhanced cancer cell survival and metastasis through a variety of physiologic roles

An interesting mechanism by which WSB1 may contribute to cancer development is through its role as an E3 ligase regulating the previously mentioned HIPK2, as well as RhoGDI2 and VHL during hypoxia [6, 9, 22, 39]. HIF-1, RhoGDI2, HIPK2, and miR-592 could be considered to form a regulatory network, linked by WSB1. Regulation of multiple, important regulatory proteins by WSB1 in the cellular response to hypoxia, may also facilitate cancer phenotypes (summarized in Fig. 3). HIF-1, a key regulatory transcription factor of the cellular hypoxia response comprised of HIF-1α and HIF1-β subunits, binds to hypoxia response elements (HRE) in the 5’ promoter region of the *wsb1* gene and induces its expression [39]. Silencing of *HIF-1α* by siRNA diminished hypoxia-induced *wsb1* expression. Hypoxia also induced significant expression of the *wsb1* gene in cancer specimens [6, 8]. In human hepatocellular carcinoma (HCC) or other cell lines, under hypoxic conditions, WSB1 binds and mediates degradation of HIPK2. This inhibits cancer cell apoptosis associated with anti-cancer treatments [39]. *wsb1* silencing through RNA interference in hypoxic HCC cell lines, rescued HIPK2. This change made HCC significantly more prone to death via chemotherapeutic agents [39].

VHL is a known tumor suppressor, which targets HIFs for degradation with its E3 ubiquitin ligase activity. HIFs, however, were not found to be directly targeted by WSB1 E3 ubiquitin ligase activity. Further studies demonstrated that WSB1 is a direct negative regulator of VHL in HEK293 cells [22]. WSB1 ubiquitinated and targeted VHL for degradation. RNAi silencing of *wsb1* in cancer cells under normoxia and hypoxia treatment stabilizes VHL, while inhibition of the WSB1 effect with MG132, a proteasome inhibitor, led to VHL survival. WSB1 and VHL were also shown to co-immunoprecipitate. Experimental data suggested that two C-terminal WD-repeats of WSB1 and the SOCS box were essential requirements for VHL ubiquitination. As the degradation of VHL through WSB1 further stabilized HIFs, this resulted in increased expression of *wsb1*--effectively creating a positive feedback loop. HEK293 cells overexpressing *wsb1* demonstrated an increase of HIF-regulated downstream genes and this correlated to increased metastatic potential through expression of *VEGFA, ALDOC, CA9,* and *SAP30*. However, as might be expected, *wsb1* expression does not significantly increase HIF1α mRNA levels.

It was recently demonstrated that osteosarcomas in both in vivo and in vitro experiments had increased metastatic potential under hypoxia [6]. Higher invasive potential, as quantitatively deduced by observation of increased cellular migration, were found in two osteosarcoma cell lines and three primary osteoblast lines. A549 and HeLa cell lines were also observed to behave similarly. In each primary cell line, hypoxia upregulated HIF-1α, which subsequently led to downstream *wsb1* upregulation. RhoGDI2 downregulation in osteosarcomas has been previously shown to activate the Rac1 and Rho pathway, which can lead to increased tumor cell metastasis potential. Hypoxia-induced WSB1 was associated with downregulation of RhoGDI2, showing physiologic relevance of this interaction in hypoxia.

While hypoxia upregulates HIF-1, and leads to upregulation of many genes manifesting the hypoxic survival response, HIF-1 also likely mediates much of the Warburg effect of altered glucose metabolism in cancer cells [30]. The published evidence now strongly suggests that in hypoxia, WSB1, through diverse mechanisms such as protection of HIF-1 through downregulation of VHL, HIPK2 and/or RhoGDI2, contributes to tumor metastasis [6, 16, 22, 26].

Dysregulation of *wsb1* expression has been documented in diverse cancer cell types, including neuroblastoma (NB), hepatocellular carcinoma (HCC), pancreatic, osteosarcoma and breast cancer cells [1, 6, 8, 23, 29, 36, 37, 39]. Copy number changes of *wsb1* can be identified in cases of neuroblastoma. Copy number of *wsb1* correlated strongly with *wsb1* expression [8]. Neuroblastoma cases of stage 4S more frequently showed gains of *wsb1* gene copy number than stage 4- cases. Increase of *wsb1* gene expression correlated with good outcome, suggesting that *wsb1* expression may have a role in the biology of good prognosis of certain neuroblastoma [8]. Through gene expression analysis, *wsb1* expression was positively correlated with high metastatic potential in melanoma, pancreatic and urinary bladder cancer cohorts [22]. Similarly, in certain breast and colon cancers, low *wsb1* expression was correlated with higher survival.

Two groups of investigators, individually studying pancreatic cancer and neuroblastoma, found that the three *wsb1* transcripts had antagonistic functions to each other [1, 36]. Interestingly, both groups discovered that tumor hypoxia modulated the splice form of the *wsb1* mRNA created [1, 36]. This was identified through the xenotransplantation of respective cancer cells in mouse, and quantifying the *wsb1* mRNA in the extract by real-time PCR (qPCR) [1, 36]. Interestingly, both groups discovered that in xenograft tumors, *wsb1* mRNA encoding for isoform 1 and 2 had been downregulated, whereas the *wsb1* mRNA encoding for isoform 3 had been upregulated [1]. Similar results had been obtained when the groups used chemotherapeutic stress agents in in vitro cancer cell cultures [1, 36]. Silencing expression of the *wsb1* transcripts through siRNA in the xenograft tumors showed reduced growth, enhanced apoptosis rate, and increased sensitivity to chemotherapeutic agents. Specifically, these effects were attributed to the expression of *wsb1* isoform 3 [1, 36]. In support of this view, the retroviral forced expression of *wsb1* isoforms 1 and 2 promoted cell growth and increased sensitivity of cells towards apoptosis. In contrast, retroviral forced expression of *wsb1* isoform 3 reduced cell proliferation and enhanced resistance to apoptosis [1].

## Conclusions

WSB1 has been identified through diverse investigations and in different experimental model systems as an important regulator of targeted protein destruction or aggregation. Protein targets of WSB1 are involved in cellular processes as diverse as thyroid hormone regulation, immune regulation and regulation of the cellular response to hypoxia. Likely through its latter effects (Fig. 3), WSB1 has been shown to be a likely regulator of abnormal proliferation and metastasis in cancer.

**Fig. 4:**
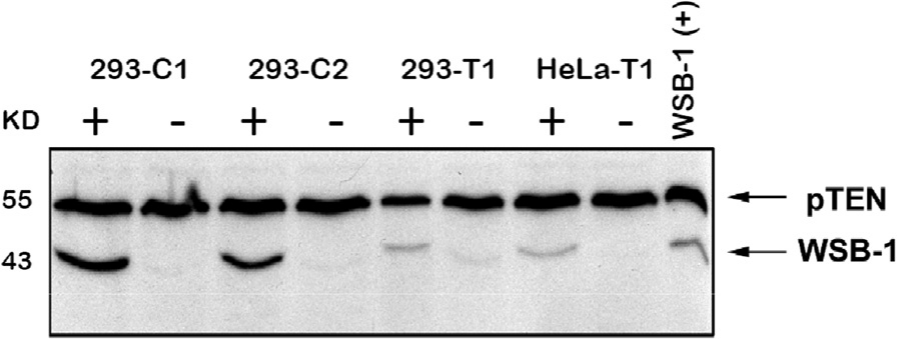
Preparation of Tet-inducible-WSB1 Cell Lines. Full length *wsb1* isoform 1 was prepared by RT-PCR and cloned into a shuttle vector. Clones were sequenced and both SNP variants C and T were identified and each was cloned in frame, into the Tet-On 3G system vector, pTRE3G (Clontech). The *wsb1* insert in pTRE3G was sequenced prior to use for transfection. *wsb1*-pTRE3G was transfected into either HeLa or HEK293 containing a stably transfected TET protein expressing construct (Clontech). Puromycin resistant clones were selected and evaluated for their inducibility with 1 mg/ml doxycycline. Figure 4 demonstrates the inducibility of *wsb1* in the presence or absence of doxycycline. Example cell lines include either WSB1 “C” variant HEK293 (2 clones), or “T” variant (one cell line) or HeLa “T” variant

**Fig. 5:**
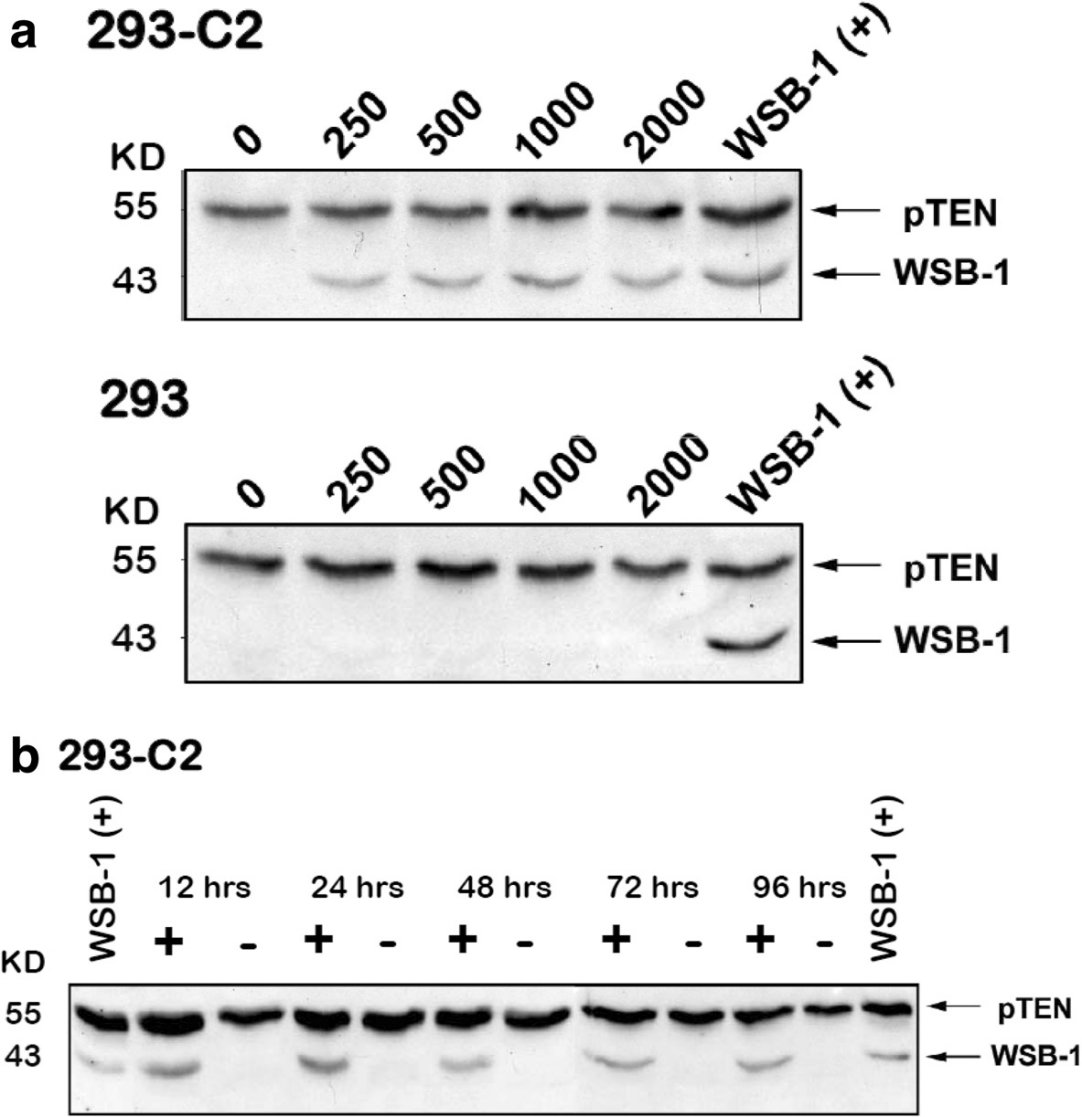
Characterization of WSB1 Induction with Doxycycline. Panel **a** HEK293 cells containing the inducible WSB1 “C” construct (293-C2) or native HEK293 cells (293) were treated with various concentrations of doxycycline (250, 500, 1000, 2000 ug/ml) for 24 h, then used for Western blotting with #604 anti-WSB1 and anti-pTEN (New England Biolabs) primary antibodies followed by detection with anti-rabbit Ig secondary antibody. Lysates from Hela cells treated with desferroximine (260 uM) were used as positive controls (WSB-1 (+)). Panel **b** HEK293 cells containing the inducible WSB1 “C” construct (293-C2) were treated with 1000 ug/ml doxycycline for various time points (in hours) and then subjected to Western blotting as for Panel A (above). Lysates from Hela cells treated with desferroximine (260 uM) were used as positive controls (WSB-1 (+))

Recently, we have constructed two different, tetracycline-inducible *wsb1*-expressing cell lines by cloning full length *wsb1* into the mCherry expression vector, pTRE3G-mCherry (Tet-On 3G, Clontech) and expressing WSB1 in either HeLa or HEK293 cells (Fig. 4). Cell lines were also created using either the “C” or “T” polymorphism variants of *wsb1*. Tet-inducible *wsb1* approximates the levels seen in hypoxia at 24 h post-induction, using an optimal concentration of doxycycline (1 mg/ml, Fig. 5). WSB1-inducible cell lines such as these, permit the study of WSB1 induced by doxycycline, without complication by the complex regulatory effects manifested through treatment of cells with hypoxia or hypoxia-mimics, which stimulate pleomorphic effects that are non-specific to WSB1 regulation. Thus, recent development of WSB1-inducible cell lines should facilitate study of this very interesting, potential oncoprotein.

## Abbreviations

D2, thyroid hormone-activating type 2 iodothyronine deiodinase; DFX, desferroximine meselyate; G-CSF, granulocyte colony stimulating factor; HCC, hepatocellular carcinoma; HIF, hypoxia inducing factor; HIPK, homeodomain-interacting protein kinase; Ihh, Indian hedgehog; LRRK, Leucine-rich repeat kinase; MBT, mid blastula transition; miR, microRNA; mRNA, messenger RNA; PD, Parkinson's disease; PTH, parathyroid hormone; PTHrp, parathyroid hormone-related peptide; RhoGDI, Rho guanosine diphosphate dissociation inhibitor; RNA, ribonucleic acid; RNAi, inhibitory RNA; SH2, src homology domain; SNP, single nucleotide polymorphism; SOCS, suppressors of cytokine signaling; VHL, von Hippel-Lindau
